# Shelf Life Extension and Improvement of the Nutritional Value of Fish Fillets through Osmotic Treatment Based on the Sustainable Use of *Rosa damascena* Distillation By-Products

**DOI:** 10.3390/foods8090421

**Published:** 2019-09-18

**Authors:** Maria C. Giannakourou, Theofania Tsironi, Ioanna Thanou, Anna Maria Tsagri, Elena Katsavou, Vladimiros Lougovois, Vasiliki Kyrana, Georgios Kasapidis, Vassilia J. Sinanoglou

**Affiliations:** 1 Department of Food Science and Technology, University of West Attica (former Technological Educational Institute of Athens), Ag. Spyridonos 28, 12243 Egaleo, Greece; ft14321@uniwa.gr (I.T.); amtsagri@gmail.com (A.M.T.); katsavouelena@gmail.com (E.K.); vloug@uniwa.gr (V.L.); vkyr@teiath.gr (V.K.); vsina@uniwa.gr (V.J.S.); 2 National Technical University of Athens, School of Chemical Engineering, Laboratory of Food Chemistry and Technology, 15772 Athens, Greece; ftsironi@chemeng.ntua.gr; 3 Department of Food Science and Human Nutrition, Agricultural University of Athens, 11855 Athens, Greece; 4 Association of Aromatic, Pharmaceutical and Fruit/Vegetable Plants Voiou Kozanis, 50100 Voio Kozanis, Greece; Gkasap@otenet.gr

**Keywords:** sea bass fillets, *Rosa damascena* distillation by-products, osmotic treatment, shelf life extension

## Abstract

The objective of this work is the comparative study of different osmotic treatments at 37 °C on the quality and shelf life of chilled sea bass fillets. Fish fillets were treated using osmotic solutions consisting of oligofructose (40%–50%–60%) and 5% NaCl with (BP/OT) and without (OT) former antioxidant enrichment by using *Rosa damascena* distillation by-products. Water activity decreased to approximately 0.95 after 330 minutes of osmotic treatment. Untreated and osmotically treated fish fillets (BP/OT) and (OT) were subsequently stored at 5 °C and their quality was evaluated based on microbial growth and lipid oxidation. Osmotic treatment extended significantly the shelf life of fish in terms of microbial growth; however, it also accelerated its lipid oxidation. The impregnation of *Rosa damascena* phenolics not only counterbalanced this negative effect, but led to a more than four-fold increase of the shelf life of sea bass, as compared to the untreated samples.

## 1. Introduction

Fish products are of great commercial interest for Greece. The Greek fisheries sector represents a primary sector of significant socio-economic importance, particularly in coastal areas traditionally dependent on fisheries. Greece has developed a large aquaculture sector, representing a major share of national seafood production. The main species farmed are two finfish (sea bass and sea bream) and shellfish (mussels). Additionally, fish fillets, with low or high fat content, are foods of superior nutritional value and increased significance for both consumers and relevant food industries. They provide approximately 16% of the protein of animal origin to the human nutrition, being also an important source of vitamins (A, B, D, E), macro and trace elements, and essential fatty acids [1]. The fish lipid fraction consists of a significant proportion of *n*-3 polyunsaturated fatty acids, e.g., C20:5, *n*-3 and C22:6, *n*-3 [2,3], with beneficial effects on human health by reducing the risk of cardiovascular and neurological diseases, cancer, coronary heart disease, and the progression of coronary atherosclerosis. In the case of sea bass, iced storage is the most commonly used preservation method, particularly for short transportation and local consumption [4]. On the other hand, fish flesh is more perishable than red meat and poultry. Fish tissue deterioration is attributed mainly to autolytic processes (due to the activity of the inherent tissue and digestive enzymes), bacterial activity (due to microbial enzymes), spontaneous chemical reactions (the oxidation of lipids/discoloration) and the loss of flesh compounds, due to fish leaching from the melting ice [5]. Chilled fish products have a short shelf life, i.e., 14–17 days at 0–4 °C for a whole fish, while the shelf life is significantly shorter for fresh fillets, i.e., 10–12 days at 0–4 °C, provided that harvesting and subsequent transport/storage is appropriately performed. Inadequate practices in sea-site and land premises lead to rapid quality deterioration and dramatically reduce the practical storage life of fish [6,7]. Thus, the development and investigation of efficient techniques in order to minimize the economic losses is necessary. These losses begin from the phase of fishing, and continue with the products’ degradation during icing, packaging, storage, transport to land storage, and their industrial processing (if applicable). Significant losses are also observed at the wholesale and retail level, as well as at the stage of final consumption, due to the indicated expiration date on packaged products [8]. In this context, fish processing industries and researchers focus on reducing losses and falsely rejected products by applying a more rational handling and distribution system. In the current commercial practice, the applicability of non-thermal processes for the conservation of such perishable products is not yet established. The advantages of these mild techniques compared to the traditional thermal processes are mainly related to the improved retention of their overall quality, nutritional, and sensory attributes. 

Osmotic dehydration (OD) is recognized as one of the most effective, mild non-thermal techniques applied on both fruit/vegetable and animal tissue; it consists of product immersion in a hypertonic solution, containing a variety of different solutes (carbohydrates, salts, etc.), each one serving a different technological purpose. Due to the different concentrations, mass transfer occurs through the selectively permeable cell membrane of the food tissue, leading to a mild dehydration of the food, which is at the same time intentionally impregnated with preselected, desirable substances [9,10]. One of the most popular commercial applications of this procedure is fish salting, where fish tissue is dehydrated and simultaneously enriched with salt, which is a process that induces significant changes on the sensory attributes and the shelf life of the processed final product [11]. 

When reviewing recent literature on OD, the use of binary osmotic solutions containing both carbohydrates and salts has many advantages, improving mass exchange, and thus promoting water loss and subsequent water activity reduction. In these cases, water activity decrease, due to salt presence, is beneficially combined with the dehydrating properties of sugar or other carbohydrates. Additionally, the presence of high molecular weight carbohydrates into the osmotic solution favors water loss and inhibits the extensive solid immersion into food tissue, which leads to a final product with similar sensory attributes as the untreated one. The most significant benefit of OD is that the appropriate preparation of the osmotic solution allows for the selective tissue modification and impregnation with antimicrobial, antioxidant, and other beneficial compounds, leading to a final product of improved nutritional, functional, and sensory attributes. Another advantage of OD is that one can optimize, after a systematic kinetic study of the tissue in question, the main process parameters (temperature, OD duration, osmotic solution content and concentration, mixing, etc.), depending on the desired characteristics of the final, processed product. There are limited studies that have indicated the advantageous application of OD on fish products [12,13,14].

Another important aspect investigated in the current research lies in the sustainable use of the valuable by-products of the essential oil production industry: ‘wastes’ that could be efficiently applied for improving fish fillet quality and significantly extending shelf life. Taking into account their organic load, ‘wastes’ from the industrial distillation of flowers (e.g., roses) and aromatic herbs in order to produce essential oils represent a serious environmental and financial hazard. The solid/liquid ‘wastes’ originating from the vegetative raw material contain high proportions of phenolic compounds, which have increased oxygen requirements, thus posing a serious problem for the regional flora and fauna. These by-products are very rich in polyphenols and are nowadays recognized as an important source of antioxidant/antimicrobial compounds that could be used to improve the functionality of a number of end products [15,16]. In Greece, *Rosa damascene* Mill (commonly known as Rose) is currently used for rose water and essential oil production, especially in the Prefecture of Western Macedonia (region of Kozani). Rose water is three times more expensive than gold, and 4000 kg of roses are approximately required to produce 1 kg of essential oil [16]. On the other hand, a negative aspect is the large amount of wastes produced from raw material distillation [12]; these by-products are currently either simply rejected to the environment or used as animal feed, being obviously under-exploited. The principal constituents of these ‘wastes’ are the solid flower residues and a big portion of liquid by-products (waste water), which are rich in organic load. These wastes, although not toxic when mildly treated by natural purification processes [17], constitute a serious environmental pollutant and lead to important inconveniences regarding their management and disposal. Therefore, it is crucial for all involved sectors to seek for new strategies to optimize and rationalize the sustainable use of these by-products, focusing on the extraction of compounds of added value. By doing so, the benefits for the industries that produce essential oils using aromatic flowers and herbs are two-fold; not only will their profits substantially increase, but additionally, their procedures will be differentiated so as to become more sustainable and environmentally-wise ‘greener’, with significantly positive effects on the regional and broader society.

Therefore, the aim of this research is dual: on one hand, the production of sea bass fillets of improved nutritional value and stability, dealing with the crucial issue of fish perishability and short shelf life and, on the other hand, the sustainable and rational exploitation of the by-products of the essential oil industry, which in the current industrial practice are being rejected and constitute a serious environmental threat. This valorization can be accomplished by their direct use from the food industry. The experimental design includes the study of the effect of the prevailing factors of the osmotic process and the enrichment with phenolic compounds exhibiting antioxidant activity, which are inherently present in the by-products, on the properties of the processed fish tissues (microbial load, physicochemical properties). This study is actually an application of the hurdle technology principles, by combining two principal preservation techniques—namely, water activity reduction (through water loss from the interior of fish fillets) and tissue impregnation with bioactive compound—to enhance its antioxidant and antimicrobial activity. The ultimate outcome could be the manufacture of a new food product of increased functionality.

## 2. Materials and Methods 

### 2.1. Fish Raw Material

Cultured sea bass (*Dicentrarchus labrax*) fillets (weight: 90 ± 10 g, capture zone: Aegean Sea, Greece) were produced by a Greek aquaculture company. Samples were transported to the Department of Food Science and Technology of the University of West Attica within 2–4 h from filleting in polystyrene containers with flake ice (0 °C) [18]. A polyethylene film was placed between layers of fillets, to avoid contact between the skin and bone sides of the fillets. Fillets were cut into rectangular slices (3 × 3 × 1 cm^3^, 10 ± 1 g) in a laminar flow hood. Osmotic solution was prepared by dissolving oligofructose (RAFTILOSE^®^, Orafti, Oreye, Belgium) and distilled water at concentrations of 40%, 50%, and 60%, adding also 5% NaCl.

### 2.2. By-Products of Rose Distillation–Infusion Raw Material

The harvest of roses took place from the middle of May 2018 up to the middle of June of the same year and lasted approximately 30 days. The harvest hours were between 05:30–9:00 in the morning, which is the period with the highest and best oil concentration. All open bloomed roses were gathered. Rose petals were kept in specific sachets and were transported as fast as possible to the distillation area (not later than an hour) [16]. After the distillation for the production of essential oil was completed, waste water was carefully collected in the distillation site and kept refrigerated (maximum storage period of two days) until delivery to our lab for analysis and further use. Waste water was checked for impurities, and after a preliminary treatment (involving coarse screening and grit removal by filtering with a 6-mm opening sieve) was kept under controlled cold storage for a maximum of one week prior to its use for fish fillets impregnation. 

### 2.3. Determination of Total Phenolic Content (TPC)

The total phenolic content (TPC) of by-products (waste water solutions) of *Rosa damascena* distillation was determined, as soon as they arrived at the Department of Food Science and Technology of the University of West Attica from the association of aromatic, pharmaceutical, and fruit/vegetable plants Voiou Kozanis (1–2 days after the distillation had taken place), according to the modified micromethod of Folin–Ciocalteu’s assay, as described in [19]. The TPC was expressed as mg of gallic acid equivalents (GAE) per L of by-product or per g of fish.

The TPC of the osmotically treated fillets was determined during the impregnation procedure, indirectly, by measuring the TPC of the osmotic solutions. All measurements were performed in triplicate.

### 2.4. Antioxidant Impregnation and Osmotic Treatment

A number of preliminary experiments were conducted in order to assess the extent of antioxidant impregnation when fish fillets were immersed into the by-products’ waste water/osmotic solution. The first attempt involved the preparation of a unique osmotic solution using waste water effluent (coming from *Rosa damascena* distillation residues) and adding oligofrustose/NaCl, and the immersion of fish samples. The second set of preliminary experiments was performed successively by the immersion of fish samples firstly in the by-products solution (BPS) for 180 min, followed by the addition of pre-weighed osmotic compounds (e.g., oligofructose and NaCl) (BPS/O) until the end of the procedure (total time: 330 min). Treatments took place at a constant temperature of 37 °C, as described in [20].

Therefore, the overall process consisted of osmotically treating fish fillets in concentrated solutions of oligofructose (40%, 50%, and 60%) and 5% NaCl at 37 °C for up to 330 min, either as a unique process (solely osmotically treated, OT) or with the previous immersion into the by-products infusion (BP/OT). A solution:sample ratio of 5:1 (*w*/*w*) was applied in order to prevent any significant dilution of the osmotic medium by water loss, which may reduce the osmotic driving force during the treatment. Beakers filled with pre-weighed osmotic solutions were placed in a water bath and brought up to 37 °C. Pre-weighed fish samples were submerged in the osmotic solution by means of a grid. At predetermined intervals, one beaker of each osmotic solution was removed from the water bath. Samples were removed from the osmotic solution, blotted gently with a tissue paper to remove the excess coating solution, and then weighed. Mass transfer kinetics were assessed by estimating the values of WL (water loss) and SG (solid gain). Each measurement was carried out in three replicates in order to take the average values and standard deviations. 

Moisture content was calculated after vacuum drying at 70 °C (Heraeus Instruments Vacutherm, ThermoScientific, Waltham, Massachusetts, USA) for 24 h. The salt content in fish flesh was calculated titrimetrically by the Mohr method using silver nitrate solution (AOAC, 1990). The water activity of fish samples was determined by an a_w_-meter (AquaLab Dew Point Water Activity Meter 4TE, METERGroup, Inc., Pullman, WA, USA), and the °Brix of the osmotic solution was measured by a hand-held refractometer (Atago, Master refractometer, Yorii, Japan). The color of fish samples was also instrumentally determined with a Handy Colour Tester, Model H-CT (Suga Test Instruments Co., Ltd., Tokyo, Japan). All measurements were performed in triplicate.

TPC impregnation was assessed as described in Section 2.2. Water loss (WL) and solid gain (SG) in fish slices were calculated according to [20], using the following equations:

WL = ((*M*_o_ − *m*_o_) − (*M* − *m*))/*m*_o_ (g of water/g initial dry matter), (1)

SG = (*m* − *m*_o_)/*m*_o_ (g of total solids/g initial dry matter), (2)

where *M*_o_ is the initial mass of fresh material before the osmotic treatment, *M* is the mass of fish samples after time *t* of osmotic treatment, *m* is the dry mass of fish after time t of osmotic treatment, and *m*_o_ is the dry mass of ‘fresh material’.

### 2.5. Shelf Life Kinetic Study

The main purpose is to design and implement a stability study of the treated samples (both OT and BP/OT), in order to compare their quality deterioration versus one of the untreated samples. The goal is to assess their shelf life, and evaluate the effect of the treatments imposed. Based on the results of the osmotic treatment and the minimum sensory alteration of fresh fish fillets, samples osmotically treated in the osmotic solution of 40% oligofructose (both OT and BP/OT) were selected for the storage study. For this study, samples were stored at controlled isothermal conditions of 5 °C in high-precision (±0.2 °C), low-temperature incubators (Sanyo MIR 153, Sanyo Electric, Ora-Gun, Gunma, Japan) for shelf life evaluation. Electronic, programmable data loggers were used for temperature monitoring in the incubators (COX TRACER^®^, Belmont, NC, USA). Sampling took place based on adequate experimental design that allows efficient analysis of the kinetic data for quality degradation.

#### 2.5.1. Microbiological Analysis

In order to determine the microbial load in the tested fish samples, 10 g of representative fish sample was homogenized with 90 mL of sterilized Ringer solution (Merck, Darmstadt, Germany) in an appropriate sterile stomacher bag for 60 s using a Stomacher (BagMixer^®^, interscience, St Nom la Bretèche, France). Then, 0.1 mL of 10-fold serial dilutions of fish homogenates were transferred and spread on the surface of appropriate culture media in Petri dishes for spoilage bacteria enumeration. Plate count agar (PCA, Merck, Darmstadt, Germany) was used for the enumeration of total viable count (incubation at 25 °C for 72 h). For the enumeration of *Pseudomonas* spp., cetrimide–fucidin–cephaloridine (CFC) agar (Merck, Darmstadt, Germany) was used (incubation at 25 °C for 48 h). Violet Red Bile Dextrose agar (VRBD, Merck, Darmstadt, Germany) was used for the enumeration of *Enterobacteriaceae* spp. using the pour plate method (incubation at 25 °C for 48 h). 

Two replicates of at least three appropriate dilutions were enumerated. Microbial growth modeling was carried out using the Baranyi growth model [21], by fitting curves using the DMFit program (http://www.combase.cc/index.php/en/). Different kinetic parameters, i.e., the rate (*k*) of the microbial growth, lag phase (Lag), and the final microbial population (*N*_max_) were estimated at the tested processing conditions.

#### 2.5.2. Total Lipid Extraction and Evaluation of Lipid Oxidation 

Total lipids (TLs) were extracted according to the Bligh and Dyer method [22], and their contents were calculated gravimetrically. Rancidity development was estimated by the thiobarbituric acid assay according to the extraction method described in [23]. The absorbance was measured at 530 nm using a digital spectrophotometer (Unicam Helios, Spectronic Unicam EMEA, Cambridge, United Kingdom). Concentrations of thiobarbituric acid reactive substances (TBARS) were calculated from a standard curve prepared by 1,1,3,3-tetraethoxypropane and expressed as mg malondialdehyde per kg of fat. Each measurement was carried out in three replicates in order to take the average values and standard deviations.

### 2.6. Statistical Analysis

The analysis of the rates of quality deterioration for the untreated, OT, and BP/OT treated sea bass fillets was performed by analysis of variance (ANOVA) at a 95% significance level using STATISTICA^®^ 7.0 (StatSoft Inc., Tulsa, OK, USA). Duncan’s multiple range test was used for the evaluation of significant differences (*a* = 0.05).

## 3. Results and Discussion

### 3.1. Characterization of the Antioxidant Capacity of Rosa Damascena By-Products 

By-products solution (BPS) from rose distillation was measured to have 1650 ± 30 mg GAE/L. In [24], an approximate value of 211.92 ± 3.10 mg GAE/g is reported for spent flowers, compared to a value of 233.56 ± 7.25 mg GAE/g for fresh flowers. On the other hand, the values reported in [25] for rose essential oil, absolute, and hydrosol (hydrodistillation product of aromatic plants, mainly used as food flavoring substances in pastries and beverages) were in the range of 839.5 ± 59.5 GAE/L, 2134.3 ± 91.4 GAE/L, and 5.2 ± 0.3 mg GAE/L, respectively. 

### 3.2. Antioxidant Impregnation and Osmotic Treatment

The one-step procedure did not show a significant antioxidant enrichment of fish during the osmosis process. Solids (including phenolics) uptake may be limited by the potential formation of a highly concentrated solute coating of the fish samples during the osmotic treatment [26]. Interestingly, maximum enrichment was found when the two procedures were performed in a sequential way; i.e., firstly, sliced samples were immersed into the by-products solution (BPS) for 180 minutes (min) (stage 1); then, the pre-weighed osmotic compounds (e.g., oligofructose and NaCl) were added into the BPS to form the final osmotic solution (BPS/O). Samples remained immersed in this solution (BPS/O) for another 150 min, so as to obtain a total immersion time of 330 min, which equals the OT procedure. This experimental set-up of BP/OT, consisting of an osmotic step with a duration of 150 min, was also based on the finding that after the first two hours of osmotic treatment, mass transfer phenomena seem to be less extensive, with WL and SG parameters obtaining an equilibrium value. The benefit of this two-stage procedure (BP/OT) has been also observed in [27], in which the authors proved the beneficial use of hypotonic (instead of hypertonic) solutions when tissue impregnation is the desired result of the immersion process. Therefore, the use of the above described sequential process was selected as the most appropriate for fish fillets enrichment with bioactive compounds.

**Figure 1:**
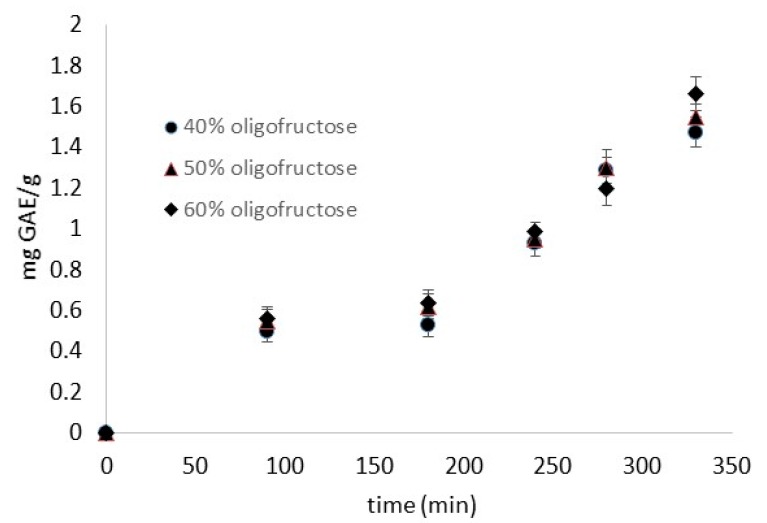
Impregnation of phenolics into fish tissue, expressed as mg gallic acid equivalents (GAE/g) (BP/OT procedure). Error bars represent the ± standard deviation of measurements. BP/OT: osmotic solutions consisting of oligofructose (40%–50%–60%) and 5% NaCl with former antioxidant enrichment by using *Rosa damascena* distillation by-products.

The impregnation of phenolics in the fish tissues during immersion in *Rosa damascena* by-product infusion, followed by the osmotic step (BP/OT procedure) is depicted in Figure 1, showing the significant enrichment of fish flesh with the desired phenolic compounds. However, the oligofructose concentration increase of the osmotic solution did not significantly affect (*p* < 0.05) phenolics impregnation, since in all cases, the final content achieved was approximately 1.5 mg GAE/g. Such a finding is considered advantageous, as the raw material reached a final phenolic content of 1500 ppm. 

**Figure 2:**
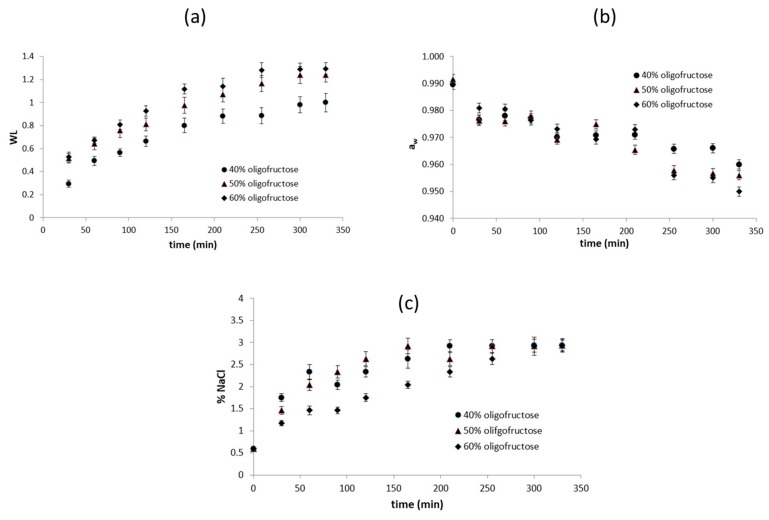
(**a**) Water loss (WL), (**b**) a_w_ decrease, and (**c**) NaCl percentage of fish samples during OT at different concentrations of the osmotic solution. Error bars represent the ± standard deviation of measurements.

Regarding mass transfer phenomena, the osmotic treatment (OT) resulted in mild moisture loss and a significant water activity reduction of fish muscle. The tested parameters, i.e., the water loss, a_w_, and NaCl percentage of sea bass samples during osmotic dehydration are illustrated in Figure 2a–c. Regarding the BP/OT process, the first stage of antioxidant impregnation within the hypotonic by-product liquid (180 min) did not seem to modify the samples’ properties, based on mass and water activity measurements (*p* < 0.05 compared to the raw material). The water loss, a_w_, and NaCl percentage change of BP/OT samples during the second step of osmotic dehydration (with a duration of 150 min) follows the same pattern as the one shown in Figure 2a–c, reaching equilibrium values close to those observed after the whole 330-min treatment (e.g., a_w_ after the overall BP/OT process, which was measured as 0.970 ± 0.003).

### 3.3. Lightness Change 

**Figure 3:**
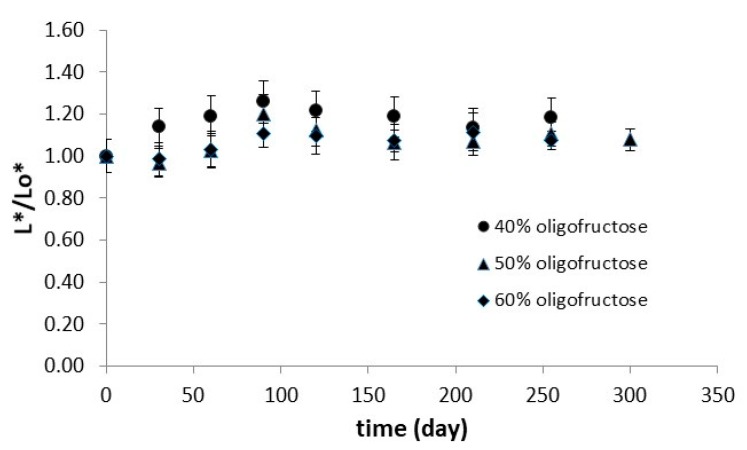
Color change (expressed as L */Lo *) during the osmotic procedure (OT procedure). Error bars represent the ± standard deviation of measurements.

Color, expressed as the relative loss of the initial lightness (L */Lo *), showed no significant (*p* < 0.05) change during the osmotic procedure (OT process) (Figure 3), and the different concentrations of the OD solution did not seem to have a significant effect on color retention. When samples were initially immersed into the BP solution up to 180 min, samples obtained a more yellowish color, and their lightness decreased (L */Lo *(at 180 min) = 1.4), which was due to the effect of the slightly colored *Rosa damascena* solution. Nevertheless, after adding the OD compounds and proceeding with the OD process (BP/OT), lightness was restored to its initial value (L */Lo *(at 330 min) = 0.99) following a similar pattern as the one shown in Figure 3 for the OT process. 

### 3.4. Shelf Life Study

#### 3.4.1. Growth of Spoilage Bacteria

Based on the results of the dehydration effect of the osmotic treatment and the sensory changes of fresh fish fillets, samples osmotically treated in the osmotic solution of 40% oligofructose, for both OT and BP/OT, were selected for the shelf life test. The growth curves of the tested spoilage bacteria (total viable count, *Pseudomonas* spp. and *Enterobacteriaceae* spp.) in fish samples (untreated, OT, and BP/OT) stored at 5 °C were fitted to the Baranyi growth model, and the growth kinetic parameters at each processing condition were calculated (*R*^2^ = 0.853 − 0.995). A small lag phase was observed in the growth of total viable count (Table 1). The microbial growth rate (k, days^-1^), i.e., the rates at the linear phase of the measured microorganisms, the initial (*N*_o_ in log cfu/g) and final population (*N*_max_ in log cfu/g), and the estimated lag phase (lag in days) are presented in Table 1. The initial total viable count of *Pseudomonas* spp. and the *Enterobacteriaceae* spp. count of untreated fish samples averaged 3.6 log cfu/g, 2.0 log cfu/g, and 1.0 log cfu/g, respectively, which is slightly below the respective values reported in the literature, indicating good hygiene practices in the fish processing plant [20,28]. The slightly higher initial microbial load after the treatments compared to the untreated samples was within the sample variability range, as indicated in the standard deviations presented in Table 1.

**Figure 4:**
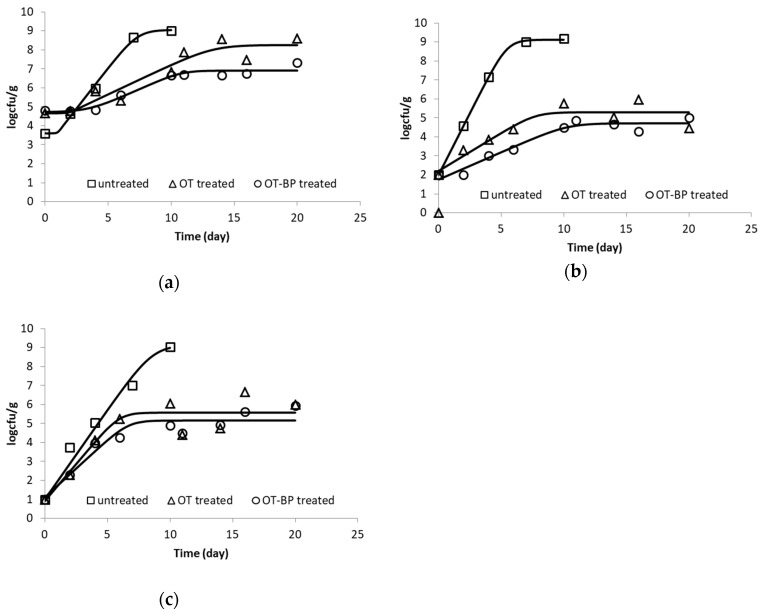
Microbial growth of (**a**) total viable count, (**b**) *Pseudomonas* spp., and (**c**) *Enterobacteriaceae* spp. for all samples stored at 5 °C (data points indicate the results of representative fish sample).

From the above results (Figure 4 and Table 1), the protective role of both the OD solutes introduced (OT process) into the fish and, more importantly, the synergistic effect of the antioxidants impregnated through the BP/OT process is obvious. This positive effect is particularly demonstrated for the growth of *Pseudomonas* spp., where the growth rate of the untreated samples was more than four-fold that of the treated fish. This finding is in agreement with previous studies, where the extract was sometimes incorporated into the icing medium of fish specimen [29,30]. In [30], researchers investigated the possible use of peanut skin (PS) and meal from dry-blanched peanuts (MDBP) as sources of phenolic compounds, and proved that these by-products, apart from their protective role against lipid oxidation, may serve even better as a source of antimicrobial phenolics. In this work, authors investigated and showed the strong antibacterial effect of these phenolic-rich extracts on the growth of both Gram-positive (*Bacillus cereus, Staphylococcus aureus, Listeria monocytogenes, Geobacillus stearothermophilus*) and Gram-negative bacteria (*Pseudomonas aeruginosa, Pseudomonas fluorescens, Salmonella Enteritidis, Salmonella Typhimurium, Escherichia coli*). The authors in [31] studied the possible quality enhancement by applying a preliminary impregnation of megrim (*Lepidorhombus whiffiagonis*) specimens in *Fucus spiralis* (a brown alga) extract. Microbial, chemical, and sensory quality was monitored in fish during chilled storage for 13 days, and an antimicrobial effect at the later stages of storage was observed, based on the comparative results on the growth of aerobes, psychrotrophs, and *Enterobacteriaceae* in megrim muscle. In [32], the authors investigated the effect of stevia phenolic compounds on lipid oxidation and microbial activity in refrigerated salmon paste, and concluded that lipid rancidity was significantly retarded, whereas microbial growth was only moderately inhibited. The authors in [33] reported significant antimicrobial activity of carvacrol and thymol on refrigerated carp fillets after immersion in 1% solutions. According to this study, the total viable count in treated carp fillets was up to 4 log cfu/g lower than the untreated samples after 12 days at 5 °C.

#### 3.4.2. Chemical Indices

**Figure 5:**
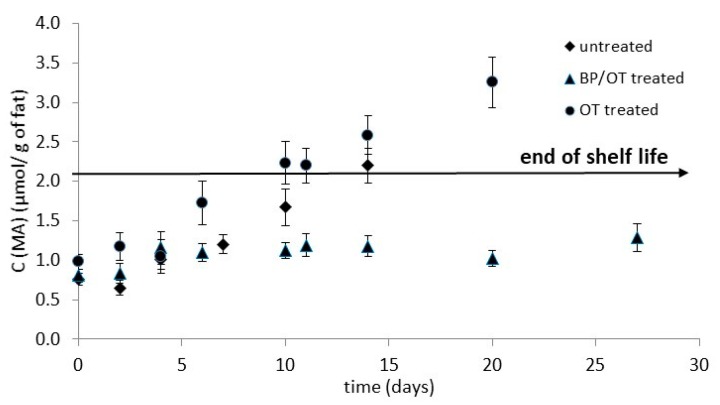
Lipid oxidation of fish fillets (expressed as concentration of malondialdehyde, µmol MDA/g of fat) during storage at 5 °C. Error bars represent the ± standard deviation of measurements.

Thiobarbituric acid (TBA) values are used to monitor the levels of secondary oxidation products. Initial malondialdehyde (MDA) levels, for all categories, were initially 0.85 ± 0.11 mg MDA/kg. OT and BP/OT samples showed significant difference in TBA values compared to the untreated samples. As expected, TBA values increased with storage in all samples (Figure 5). Control slices reached a value of 1.7 mg MDA/kg after 10 days, OD after 6 days, and BP/OT remained at lower levels, even after 27 days of storage at 5 °C. Therefore, immersing fish samples in OD solution (without the addition of antioxidant compounds from the by-product infusion) and isothermal treatment for 330 min at 37 °C seems to accelerate their oxidation. This negative effect may be attributed to the water activity decrease, combined with the negative effect of elevated temperatures, which are factors that are well known to trigger fish tissue lipid oxidation. Karel (1980) [34] proposed a possible mechanism of the “water effect”, according to which, “water content is a major factor affecting the rate of lipid oxidation in foods and several hypotheses have been advanced to explain water’s retarding effect on lipid oxidation.” When addressing the possible reasons for this water effect, one should consider the complex free-radical chain reactions of lipid peroxidation and the possible influence of the initiation and/or termination step. As far as the effect of temperature is concerned, it has been demonstrated that lipid oxidation rates increase with elevated temperatures [35], and an explanation of the prevailing mechanisms is detailed in [36] by describing the reactions occurring during the successive stages of initiation, propagation, termination, and inhibition. Furthermore, the applied osmosis temperature may accelerate the degradation of triglycerides and phospholipids by activating lipases and phospholipases [37], producing free fatty acids (FFA) that are more susceptible to oxidation [38].

On the other hand, impregnation with antioxidants within the combined procedure of BP/OT seems not only to counterbalance this negative impact on lipid oxidation, but also to retard significantly the oxidation process, leading to very low levels of MDA/g of fat, even after almost 30 days of storage. This protective effect of antioxidants in fish muscle samples has been discussed extensively in recent literature [31,39]. It has been reported that plant-based phenolic extracts (by-products out of cabbage leaves and banana peels) can be used to inhibit lipid oxidation and prolong the shelf life of fish products [40]. The authors justified this protective effect on the interaction of the phenolic extracts (having an antioxidant power) with the free lipid-peroxy or lipid-oxy free radicals (products of lipid oxidation), leading to the prevention of their further self-breakdown. In another study [41], the authors reported the advantageous use of *Stevia rebaudiana* stem waste as an edible plant-based antioxidant to inhibit fish oil oxidation. It has been also demonstrated that rosemary extract may reduce lipid oxidation and preserve the content of ɑ-tocopherol in fish oil-enriched cow milk stored at low temperatures [42]. In [43], the authors evaluated the role of mint extract (ME) and citrus extract (CE) on lipid oxidation and biochemical changes of Indian mackerel during frozen storage; the preservative effect of the plant extract, in terms of TBARS formation inhibition, was observed, especially for the samples treated with mint. 

#### 3.4.3. Shelf Life Determination

Regarding shelf life determination, the total lipid content of the studied cultured sea bass samples was determined, giving an average fat content of 5.2%. Assuming as acceptable a lipid oxidation level at the end of the shelf life being at 8 mg MA/kg fish flesh [44], and the estimated limit of 2.1 µmol MA/g of fat, one could graphically roughly estimate fish shelf life after the alternative treatments, namely 14 days for the untreated samples, 10 days for the OT-treated samples and a significantly longer time period (hardly distinguished by the plot) for the BP/OT-treated samples. Similarly, if the acceptability limit was set based on microbial spoilage and assumed to be equal to total viable count, reaching a level of 10^7^ cfu/g (estimated when fish was organoleptically rejected by a preliminary sensory test and also reported in [25], for untreated (control) and OD-treated *Sparus aurata* fillets) with the incorporation of antimicrobial agents in the osmotic solution; then, the fish fillet shelf life could be estimated using the following equation (Equation (3)), according to [14,28]:

*t*_SL_ = *t*_lag_ + (log*N*_t_ − log*N*_o_)/*k* (3)

where *N*_t_ and *N*_o_ are the final (limit of acceptability) and the initial microbial population, *t*_lag_ is the lag phase, and *k* is the relative growth rate (Table 1). Therefore, their shelf life was found to be 5 days for the untreated samples, 9 days for the OT treated samples, and 11 for the BP/OT-treated ones. 

According to [28], the addition of carvacrol and glucono-δ-lactone in a maltodextrin/NaCl osmotic solution posed additional hurdles to microbial growth in gilthead sea bream fillets, especially at low storage temperatures (0–5 °C). In [45], the authors investigated the antioxidant and antimicrobial effect of rosemary essential oil EO and extracts by incorporation in a carboxyl methyl cellulose-based edible coating of smoked eel. In this study, the addition of the extract at 200–800 ppm (total phenolic basis) in the coating provided significant antioxidant protection, which increased with concentration. However, the antimicrobial activity of EO and the extracts was moderate, with the 800 ppm concentration of the extract showing the best results in terms of total viable count, as well as *Pseudomonas* spp. and *Enterobacteriaceae* growth reduction. Similar observations have been reported in [46], as the authors studied the antimicrobial and antioxidant effect of *Satureja thymbra* extracts and EO incorporated into an edible coating for gilthead sea bream fillets, which resulted in up to 35% shelf life extension and an approximately three-fold reduction of peroxide values during refrigerated storage. The addition of *Rosmarinus officinalis* in combination with vacuum packaging resulted in a significant inhibition of microbial growth and chemical changes (biogenic amines production and lipid oxidation) in swordfish steaks [47].

## 4. Conclusions

The objective of the present study was to evaluate the effect of osmotic dehydration with and without antioxidants from *Rosa damascena* distillation by-products on the quality characteristics and the shelf life of sea bass fillets. Osmotic treatment resulted in a significant improvement in quality stability during refrigerated storage, in terms of microbial spoilage, whereas it was proved to trigger lipid oxidation. On the other hand, the combined osmotic process with the addition of impregnated antioxidants was by far the most effective treatment for the preservation of sea bass fillets, regarding all the indices studied. Considering that fish is characterized by a low content of endogenous antioxidants compared to other food items [38], one could conclude that this study could serve as a first investigation on the production of fish fillets of improved nutritional value and stability, combining the sustainable and rational exploitation of the by-products of the essential oil industry. In this context, it has been shown that the specific by-products are an excellent source of natural antioxidants and can be extensively used for the development of added-value functional foods as high-quality ingredients. At the same time, the proposed fish processing method shows the potential to increase the shelf life and thus benefit the aquaculture via economic incomes and the consumers by improving the fish quality and nutritional value [40]. Further studies are necessary in order to investigate and optimize fish processing with OD, in order to maximize the preservative effect, and at the same time determine the impact of these treatments on other sensory attributes, aiming at maintaining the original sensory properties of fish fillets, i.e., color, odor, and taste. 

## Figures and Tables

**Table 1:** Growth rates (*k* in days^−1^), initial (*N*_o_ in log cfu/g) and final population (*N*_max_ in log cfu/g), and lag phase (Lag in days) of total viable count, *Pseudomonas* spp. and *Enterobacteriaceae* spp. of sea bass slices stored at 5 °C

	Total Viable Count				*Pseudomonas* spp.				*Enterobacteriaceae* spp.			
	*k*	*N*_o_	*N*_max_	Lag	*k*	*N*_o_	*N*_max_	Lag	*k*	*N*_o_	*N*_max_	Lag
untreated	0,85 ± 0.083 ^a^	3.6 ± 0.7	9.0 ± 0.2 ^a^	0.9 ± 0.3 ^a^	1.30 ± 0.023 ^a^	2 ± 0.3	9.1 ± 0.1 ^a^	-	0.94 ± 0.23 ^a^	1 ± 0.2	9.1 ± 1.0 ^a^	-
OT	0,31 ± 0.081 ^b^	4.6 ± 0.8	8.3 ± 0.4 ^b^	1.8 ± 0.5 ^b^	0.40 ± 0.13 ^b^	2 ± 0.5	5.3 ± 0.3 ^b^	-	0.78 ± 0.24 ^a^	1 ± 0.3	5.6 ± 0.3 ^b^	-
BP/OT	0,30 ± 0.097 ^b^	4.7 ± 0.8	6.9 ± 0.1 ^c^	3.4 ± 1.8 ^b^	0.29 ± 0.049 ^b^	2 ± 0.4	4.7 ± 0.2 ^c^	-	0.61 ± 0.14 ^a^	1 ± 0.3	5.2 ± 0.2 ^b^	-

Mean values ± standard error based on the statistical variation in the kinetic parameters of the Baranyi growth model—regression analysis). OT: samples osmotically treated in osmotic solutions consisting of oligofructose (40%–50%–60%) and 5% NaCl, BP/OT: samples immersed in *Rosa damascena* distillation by-products before being osmotically treated in osmotic solutions consisting of oligofructose (40%–50%–60%) and 5% NaCl. a–c: Different superscripts in the same column indicate significant differences (*p* < 0.05).
